# Effectiveness of a Mindfulness and Self-Compassion Standard Training Program versus an Abbreviated Training Program on Stress in Tutors and Resident Intern Specialists of Family and Community Medicine and Nursing in Spain

**DOI:** 10.3390/ijerph181910230

**Published:** 2021-09-28

**Authors:** Juan Carlos Verdes-Montenegro-Atalaya, Luis Ángel Pérula-de Torres, Norberto Lietor-Villajos, Cruz Bartolomé-Moreno, Herminia Moreno-Martos, Luis Alberto Rodríguez, Teresa Grande-Grande, Rocío Pardo-Hernández, Benito León-del-Barco, Mirian Santamaría-Peláez, Luis A. Mínguez, Josefa González-Santos, Raúl Soto-Cámara, Jerónimo J. González-Bernal, on behalf of the MINDUUDD Collaborative Study Group

**Affiliations:** 1 Family and Community Medicine Teaching Department of Burgos, 09006 Burgos, Spain; juancarlosverdesm@yahoo.es (J.C.V.-M.-A.); tgrande@saludcastillayleon.es (T.G.-G.); 2 Multi-Professional Teaching Unit for Family and Community Care of Cordoba, Healthcare District of Cordoba and Guadalquivir, Institute Maimonides Research Institute Cordoba (IMIBIC), Reina Sofía University Hospital, University of Cordoba, 14001 Cordoba, Spain; langel.perula.sspa@juntadeandalucia.es; 3 Family and Community Medicine Teaching Department of Jaen, 23007 Jaen, Spain; norberto.lietor.sspa@juntadeandalucia.es; 4 Family and Community Medicine Teaching Department of Zaragoza Sector 1, 5018 Zaragoza, Spain; cbartolomem@hotmail.com; 5 Multi-Professional Teaching Unit for Family and Community Care of Almería, 04009 Almería, Spain; partaloa@gmail.com; 6 Family and Community Medicine Teaching Department of Ponferrada, 24400 León, Spain; lalberto.rodriguez.sspa@juntadeandalucia.es; 7 Department of Health Sciences, University of Burgos, 09001 Burgos, Spain; rph1001@alu.ubu.es (R.P.-H.); rscamara@ubu.es (R.S.-C.); jejavier@ubu.es (J.J.G.-B.); 8 Department of Psychology, Faculty of Teacher Training College, University of Extremadura, 10071 Caceres, Spain; bleon@unex.es; 9 Department of Educational Sciences, University of Burgos, 09001 Burgos, Spain; laminguez@ubu.es

**Keywords:** stress, PSQ, mindfulness, MBSR, primary care, tutors, resident intern specialists

## Abstract

Stress is one of the most common problems among healthcare professionals, as they are exposed to potentially stressful and emotionally challenging situations in the workplace. Mindfulness-based stress reduction (MBSR) training programs have been shown to decrease stress. The objective of this study was to compare the effectiveness of an abbreviated 4-weeks MBSR training program in relation to a standard 8-weeks one on the stress levels. A controlled and randomized clinical trial was designed, in which 112 tutors and resident intern specialists in Family and Community Medicine and Nursing of six Spanish National Health System teaching units (TUs) participated. Participants included in the experimental groups (EGs) received a MBRS training program (standard or abbreviated), while control group (CG) participants did not receive any intervention. The stress levels were assessed by the Perceived Stress Questionnaire (PSQ) in three different moments during the study: before, immediately after, and 3 months after the intervention. Adjusted covariance analysis (ANCOVA), using pretest scores as the covariate, showed a significant reduction in stress (F_(2,91)_ = 5.165; *p* = 0.008; η^2^ = 0.102) in the post-test visit, attributable to the implementation of the standard training program, but without the maintenance of its effects over time. No significant impact of the abbreviated training program on stress levels was observed in the intergroup comparison. A standard 8-weeks MBSR training program aimed at tutors and resident intern specialists in Family and Community Medicine and Nursing produces significant improvements in stress levels compared with the abbreviated intervention and no intervention. New studies about abbreviated training programs are needed to provide effective treatments which improve well-being of these professionals.

## 1. Introduction

Stress is the product of a person’s interrelation with their context, and it appears when a person values that the situation exceeds their resources for action. Under these conditions, the person uses multiple cognitive and emotional efforts that determine their response to the environment and particular coping strategies [1]. Stress may occur when works demands and pressures exceed the worker’s adaptive response and resources to control the situation, or when the worker perceives an imbalance between invested efforts and expected rewards [2,3].

Stress is one of the most common problems among healthcare professionals [4], as they are exposed to potentially stressful and emotionally challenging situations in the workplace [5]. The perceived stress levels by the healthcare professional can be increased by different factors. Some of them are inherent to the job, such as the long working hours, the unpredictability of work, the contact with suffering, pain and death, the high cognitive and emotional demands, or the support for families, while others are external to the job, such as the high workload, the staffing shortages, the psychosocial environment of the work, the existence of users increasingly demanding solutions to their needs and health problems, the greater need for knowledge, the insufficient time for continuous training and retraining, or the perception of lack of support from managers [6,7,8]. In the same way, the support of work colleagues, help with the workload and emotional support, access to professional support, effective leadership strategies and the learning environment act as protective factors against the stress of healthcare professionals [9].

In addition, in the current epidemiological situation derived from the COVID-19 pandemic, the appearance of stressful situations has been increased due to fear of being infected, possibility of transmitting the disease to their relatives, confinement and, in some cases, voluntary isolation [10,11]. However, the evidence shows that the appearance of stress is not entirely about objective exposure and danger, but more about a person’s health subjective cognitive–emotional elaboration of a given situation and beliefs models [12,13]

Previous studies have shown that stress amongst healthcare occupations have been associated with physical and mental health problems, such as alteration of high-level cognitive functions, specifically memory and attention, anxiety, depression, diabetes mellitus, heart disease, hypertension, insomnia, or obesity. This situation can have important and significant repercussions on the personal and professional life of the worker, such as increased medical errors, reduced job satisfaction, decreased patient satisfaction, increased work absenteeism, substance abuse, disruption to personal relationships, as well as a variety of other mental health problems [14,15,16,17,18,19].

In Spain, the National Health System has adopted the residence system in the specific area of post-graduate healthcare professionals teaching. In this training process, resident intern specialists are expected to assume responsibilities progressively in different areas of competence. In this organization, a key figure is the tutor, who is a professional with a minimum amount of experience in patient care and who selflessly and voluntarily supervises the activities of the resident intern specialist [20,21]. Therefore, tutors and resident intern specialists share expectations and responsibilities of teaching and learning with the clinical practice. The high demands for care to resident intern specialists, as well as the high workload of tutors, increase the risk of suffering stress in this group of healthcare professionals [22].

In order to reduce the physical and psychological consequences of stress in healthcare professionals, it is necessary to implement measures regarding work conditions and work organization, as well as to provide professionals with the necessary tools promoting self-care to help them cope with reality through emotional self-regulation [23,24].

Mindfulness training programs have proved to be an effective technique in reducing perceived stress, and improving empathy and emotional management in health professionals, with the maintenance of their effects in the medium–long term [25,26,27]. However, its implementation in Spanish territory has been very uneven in recent years, with a very small number of Primary Care professionals who know techniques to improve self-awareness and psychological well-being [28]. Mindfulness is considered as a third generation therapy, defined by Kabat-Zinn as “the ability to pay attention on purpose in the present moment, without judgment, to the development of one’s own experiences moment to moment” [29]. This practice is based on training the self-regulation of attention and consciousness to improve the control of mental processes, increasing well-being [30,31].

Related to mindfulness, self-compassion is defined as “being open to and moved by one’s own suffering, experiencing feelings of caring and kindness toward oneself, taking an understanding, non-judgmental attitude toward one’s inadequacies and failures, and recognizing that one’s experience is part of the common human experience” [32]. This aspect is relevant among healthcare professionals, to the extent that they have to know how to respect and accept themselves to then increase their capacity to feel and show empathy and compassion towards others [33], generating feelings of closeness and affection [34,35,36]. It is a resilience factor linked to less stress and feelings of exhaustion, psychopathology, and greater well-being [37]. Self-compassion is often included in mindfulness training programs of healthcare professionals in order to improve their relationship and communication with the patient [38,39]. The combination of both practices, mindfulness and self-compassion, has proven to be an effective intervention in healthcare professionals with high levels of stress, requiring new studies to analyze its long-term effects [40,41,42,43,44]. The mindfulness-based stress reduction (MBSR) training program, developed in 1979 by Jon Kabat-Zinn at the University of Massachusetts (USA), consists of eight 2.5-h group sessions a week along with 45 min a day of practice at home for six days a week [45]. A beneficial effect in mental and physical health among different clinical population has been shown in different meta-analysis [24,46]. This program has been designed to grasp the principles of self-regulation and develop skill and autonomy in mindfulness practice for participants. It requires a high level of adherence and considerable commitments of time to complete the training. However, the circumstances of some groups exclude them from participating in this standard form [47,48]. Different studies have tried to reduce the implementation time of these programs, in order to increase their viability while maintaining their effectiveness. In a meta-analysis of 15 studies, the abbreviated 4-week MBSR training program was as effective as the standard 8-week one in improving the psychological functioning of healthcare professionals [49].

New research is needed to support the effectiveness of the abbreviated mindfulness and self-compassion training programs in healthcare professionals, especially in tutors and resident intern specialists in Family and Community Medicine and Nursing, in order to recommend their inclusion in the curricular programs of the specialty and continuous training. Therefore, the objective of this study was to compare the effectiveness of an abbreviated 4-week MBRS training program in relation to a standard 8-week one on the perceived stress levels in tutors and resident intern specialists in Family and Community Medicine and Nursing in Spain. The hypothesis of this study was that the shortened four-session program of mindfulness and self-compassion is at least as effective as the standard eight-session program to improve the levels of perceived stress of tutors and resident specialists in Spain

## 2. Materials and Methods

### 2.1. Design and Setting

An open-label, pragmatic, non-inferiority, multicentre, controlled and randomized cluster clinical trial was designed, grouped in three parallel arms: a control group (CG) and two experimental groups (GE1 and GE2).

The study protocol has been previously published [50] and registered in the ClinicalTrials.gov website, supported by the United States National Library of Medicine, with reference number NCT03629457.

In this manuscript, the results about stress are presented as part of the primary outcomes of the clinical trial [51].

### 2.2. Study Participants and Recruitment

As was published previously in the study protocol [50], the study population consisted of 802 Primary Care professionals, tutors (*n* = 297), and resident intern specialists in the Family and Community Medicine or Nursing (*n* = 595), from 6 Teaching Units (TUs) of the Spanish National Health System of different dimensions, according to the population density of each territory, distributed across the geography: Almeria (*n* = 147), Burgos (*n* = 64), Córdoba (*n* = 256), Jaén (*n* = 185), Ponferrada (*n* = 63) and Zaragoza Sector I (*n* = 87). Professionals who had previously attended a mindfulness training course or workshop of at least 4 weeks, those who were active mindfulness practitioners, those who were on prolonged sick leave during fieldwork, or those who had mental disorders discouraging the development of the interventions were excluded.

Participants were contacted and recruited through the usual communication channels existing in each of the 6 TUs. After explaining the objective and methodology of the study as well as its voluntary nature, the professionals were invited to participate in it, having to sign the commitment form and the informed consent in case of acceptance.

### 2.3. Sample Size

Sample size estimation was carried out considering the potential modification of the mean score of the Five Facet Mindfulness Questionnaire (FFMQ), as the main variable of this clinical trial. Considering given alpha and beta risks of 0.05 and 0.20, respectively, in bilateral contrast, and a standard deviation (SD) of ±20, 114 participants were required (38 for each of the groups) to detect a minimum difference ≥ 15 points in the FFMQ between EGs and CG. A predicted follow-up loss rate of 25% was also assumed [51,52]. These calculations were based on the results obtained in a previous study [41]. In addition, when the sample size was calculated, the effect of the study type or its design was also taken into account. To achieve the same power between the intergroup and intra-group variance, a multiplying factor was applied [53]. With an intra-cluster correlation coefficient of <0.05, the most common in clinical trials developed in Primary Care [54], and an effect of the design type of 1.7, the sample should be made up of 132 professionals, 22 for each TU and 44 in each comparison group.

### 2.4. Procedure and Randomisation

**Figure 1:**
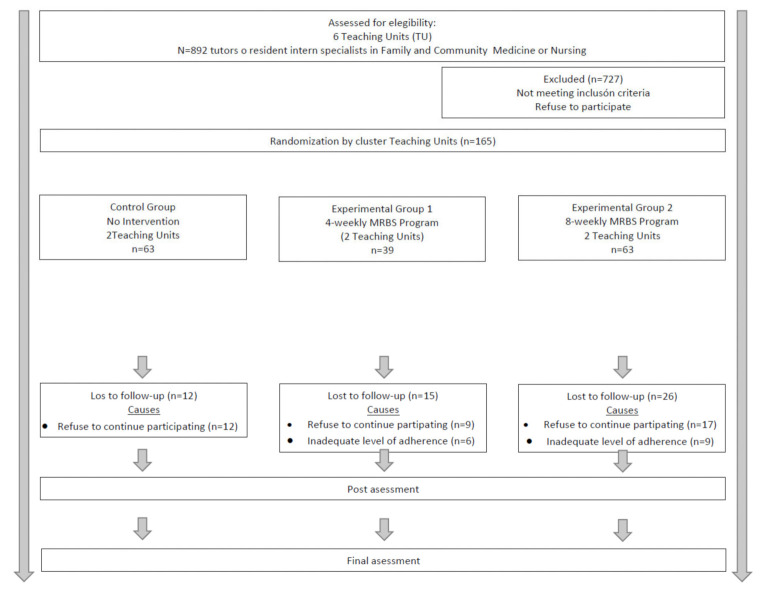
Flow-chart of the cluster-randomized trial and intervention procedure.

The study variables were measured in all participants at an initial or baseline evaluation visit (pretest), one week before the start of the sessions in the EGs. Subsequently, the final evaluation visit (post-test) was carried out at 4 weeks for the participants of EG1 and at 8 weeks for those of EG2 and the CG. In turn, the EG participants were reassessed 3 months after the end of the interventions at the follow-up visit, to verified the maintenance of their effects over time. (Figure 1).

Each TU was considered as a different and independent cluster, randomly assigned to the CG (2 TUs) or one of the two EGs (4 TUs). EG1 participants were included in a standard training program of mindfulness and self-compassion; while EG2, in an abbreviated one. Furthermore, the participants from each TU were stratified according to the type of professional (66 tutors versus 66 resident intern specialists). (Figure 1).

The characteristics of the interventions impeded the blinding of the participants. With the aim of minimizing the possible cross-contamination between groups, the training sessions and the evaluation visits, as well as the statistical analysis, were conducted by different researchers. Furthermore, clear instructions were provided to all participants regarding not disclosing during the assessment visits the group to which their TU had been assigned.

### 2.5. Intervention

Participants in the two EGs were included in a MBSR training program [41,55], complemented with practices of the Mindful Self-Compassion (MSC) program [34,35,36]. The sessions to be tested were adapted to the characteristics of each group, differing only in their duration and in the time dedicated to the different tasks by the participants [41,56]. In GE1, participants received an abbreviated training program whose format was 4 weekly sessions of 2.5 h duration, having to practice for 15 min a day at home. In GE2, the format of the standard training program was 8 weekly sessions of 2.5 h duration together with 30 min daily practice at home. The sessions was hold in group, altering moments of silence with others of collective exploration on the best strategies to address complex and difficult situations, always looking for its practical application in the personal and/or professional fields of the participants. The contents of the sessions were oriented to the knowledge of mindfulness, the perception of reality, stress and emotional management, the use of mindful communication, resilience, self-care, or time management, as well as their integration into daily life. In the previously published study protocol, the activities and tasks developed in each of the sessions have been detailed [50]. The sessions were unified in the different TUs and taught by the same instructors with university accreditation, following standardized and uniform methodological criteria.

On the other hand, no type of intervention was applied to the CG participants to be able to compare with the real situation of the health workers who do not participate in stress reduction activities. In addition, they were asked to pledge not to participate in the practice of any session of mindfulness or mediation techniques during the study period. After the completion of the fieldwork, the possibility of receiving the sessions of the abbreviated training program were offered to them.

### 2.6. Main Outcomes

The main outcome of the study was the perceived stress level of the participants, which was assessed in the pretest, post-test, and follow-up visits.

To understand how different situations could affect the feelings and stress of the participants, the Perceived Stress Questionnaire (PSQ) was used. This instrument, prepared by Levenstein in Italian and English, which was validated for the Spanish population by Sanz-Carrillo et al. [56], evaluates six factors related to stress: tension–instability–fatigue, social acceptance of conflicts, energy and fun, overload, self-fulfillment satisfaction, fear, and anxiety. Its 30 items refer to the frequency at which each stressful event has occurred in two different times: the month immediately before and the last year. It is measured by a four-point Likert-type scale, from 1 “almost never” to 4 “almost always”. Individual total score is expressed as the sum of the score of all factors, and ranges from 30 to 120 points, with higher scores indicating higher perceived stress. Its internal consistency, in the present study, is 0.946 for annual scores and 0.927 for monthly ones [56].

To assess the participants’ adherence to the training programs, attendance at the face-to-face sessions was continuously followed-up. In addition, a daily record of the practices at home was requested to them, which had to be shown to the instructor in each session for supervision. Participants who had completed at least 3 of the 4 sessions in GE1 or 6 of 8 in GE2 were considered to have an adequate adherence, and their data were included in the subsequent statistical analysis.

In order to control for potential predictors or confounding effects, sociodemographic variables such as age, sex (male or female), profession category (physician or nurse), type of professional (tutor or resident intern specialist), work center (hospital or health center), the number of years of experience as tutor, and time working in the Spanish National Health System or TU were collected during the initial evaluation visit.

### 2.7. Data Collecption Procedure, Data Management and Monitoring

Measurement and data collection in the evaluation and follow-up visits were conducted by researchers who had been previously trained for the task. This person was not the researcher in charge of making the randomization process or the subsequent statistical analysis of the data. A unique alphanumeric code was assigned to each study participant in order to identify the data collected in the different evaluations. For this purpose, a database was created, which could only be accessed by the researchers who worked in the study. Double data entry procedure was used for all questionnaires to keep the error rate as low as possible. The cleaning and clearing process in the database at the end of the study was carried forward by the principal researcher.

### 2.8. Ethical Considerations

The Clinical Research Ethics Committee of the Reina Sofía Hospital of Córdoba (Spain) approved the protocol of this clinical trial, with reference number 3845. The written and signed informed consent was provided by each participant, according to the general recommendations of the Declaration of Helsinki. All participants were informed about the objective of the study as well as the risk and benefits. The data obtained were not used for other aims than those expressed in the written informed consent or transferred to third parties outside the study. The confidentiality of the participants’ data was guaranteed at all times in accordance with the provisions of Organic Law 3/2018, of 5 December, on Personal Data Protection and Guarantee of Digital Rights, the Law 14/2007, of 3 July, on Biomedical Research, and the EU Regulation 2016/679 of the European Parliament and of the Council, of 27 April 2016, on the General Data Protection of Natural Persons with regard to the Processing of Personal and Free Circulation of such Data.

### 2.9. Statistical Analyses

An intent-to-treat analysis was performed in order to control the effects of non-random dropouts and losses. The data from the last observation carried out were attributed to dropouts or withdrawals. The characteristics of the study sample population were presented as mean and standard deviation (DS) for the quantitative variables and as frequency distribution and percentages for the categorical variables. The quantitative variables were checked for normal distribution using the Kolmogorov–Smirnov test, and all of them were considered normally distributed. To evaluate the comparability in the baseline visit between the three study groups, the chi-squared test or the Student’s t test for independent samples were used. The effects of the MBSR training programs on the outcomes measures were evaluated using the analysis of variance (ANOVA) test to compare the means between the three groups. The changes in the stress levels in each group at the final or follow-up visits with respect to the baseline visit were analyzed using the ANOVA test for paired data. The Bonferroni test was used for the post-hoc analysis. The Mauchly´s W test was calculated in order to determine the presence or absence of sphericity, performing the Greenhouse–Geisser correction if necessary. Cut-off values for Cohen’s d’s were d < 0.19 = trivial effect size (T); 0.20 < d < 0.49 = small effect size (S); 0.50 < d < 0.79 = medium effect size (M); d > 0.80 = large effect size (L) [57]. A covariance analysis (ANCOVA), using the pretest scores of the dependent variables as covariate and the intervention groups as a fixed factor, was performed in order to eliminate the effect attributable to variables not included in the design and, therefore, not subjected to experimental control, from the dependent variables (post-test and follow-up scores). Statistical analysis was performed with SPSS^®^ 25.0 (IBM Corporation, Armonk NY, USA) for Windows^®^^®^ and MLwiN version 3.0 software (Centre for Multilevel Modelling, University of Bristol, Bristol, UK, 2019). Statistical significance was considered if *p* < 0.05.

## 3. Results

### 3.1. Baseline Characterists of the Study Participants

The initial study sample consisted of 165 participants, distributed as follows: 63 in the CG, 39 in the EG1, and 63 in the EG2. During the fieldwork, there were 54 losses: 38 because the subject refused to continue participating in the study and 15 due to an inadequate level of adherence to the training program. Therefore, the final study sample consisted of 112 participants, who were included in the subsequent analysis, with 51 in the CG, 24 in the EG1, and 37 in the EG2. (Figure 1).

Table 1 summarizes the baseline sociodemographic characteristics of participants according to the study group. The mean age of the participants was 40.61 years (DS ± 12.61) and most of them were women (*n* = 86, 76.79%). The physician was the most represented professional category (*n* = 95; 84.82%), with 84.82% of the participants working in Primary Care (*n* = 95). The mean work experience was 12.88 years (SD ± 13.15). The tutors and resident intern specialists were distributed equally in the sample (50 versus 62). At baseline, statistically significant differences were found between the three groups in age, professional type, and work experience.

### 3.2. Mindfulness and Stress Intervention

In the pretest inter-group comparisons, the absence of significant differences (*p* ≥ 0.05) in the PSQ scores showed equivalence between the CG/EG1/EG2 as a basis for comparison (Table 2). In the post-test inter-group comparisons, significantly lower scores were obtained in the variables PSQ-Tension, PSQ-Social, PSQ-Everyfun, PSQ-Satisfaction, and PSQ total score. Bonferroni pairwise comparisons showed that these differences were established between CG and EG2 (PSQ-Tension, *p* = 0.008; PSQ-Social, *p* = 0.016; PSQ-Everyfun, *p* = 0.008; PSQ-Satisfaction, *p* <0.001; PSQ total score, *p* = 0.043). There were no significant differences between the CG and EG1, or between EG1 and EG2. In the follow-up inter-group comparisons, significant scores were only obtained in the PSQ-Satisfaction factor. Bonferroni pairwise comparisons showed that these differences were established between the CG and the EG2 (*p* = 0.019).

The intra-group comparisons showed a significant reduction between the pretest, post-test, and follow-up scores within the CG and EG1 in the variable PSQ-Everyfun with significant but minimal effect sizes (η^2^ = 0.130 and η^2^ = 0.190, respectively). Bonferroni pairwise comparisons showed that these differences were established between the pretest and post-test scores (*p* = 0.015 and *p* = 0.025, respectively). On the other hand, within EG2, significant reductions were established between the variables PSQ-Tension, PSQ-Everyfun, PSQ-Satisfaction, PSQ-Fear, and PSQ total score. Moderate effect sizes were obtained in the variables PSQ-Everyfun and PSQ-Satisfaction (η^2^ > 0.292). Bonferroni pairwise comparisons showed that the differences were established between the pretest and post-test scores (PSQ-Tension, *p* = 0.019; PSQ-Everyfun, *p* = 0.001; PSQ- Satisfaction, *p* < 0.001; PSQ total score, *p* = 0.010). Differences were only observed between the pretest and the follow-up in the variable PSQ- Satisfaction (*p* = 0.017). There were no significant differences between the post-test and follow-up scores (Table 3).

Using the pretest scores of the dependent variables as covariates, the ANCOVA post-test showed significant differences between the EGs and CG in the variables PSQ-Tension, PSQ-Social, PSQ-Everyfun, PSQ-Satisfaction and PSQ total score, confirming the intergroup comparisons in the post-test evaluation. Therefore, these significant differences, mainly in EG2, could be attributed to the MBRS training program. In this analysis, no significant differences were found between the groups in the follow-up evaluation (Table 4).

## 4. Discussion

In this study, the effects and potential benefits of an abbreviated and a standard training program in mindfulness and self-compassion on stress levels in tutors and resident intern specialists in Family and Community Medicine and Nursing have been analyzed and compared. In the participants who received the standard MBRS training program, an improvement in PSQ total score, as well as PSQ-Tension, PSQ-Social, PSQ-Everyfun and PSQ-Satisfaction subscale scores, was observed immediately after the intervention, but without the maintenance of its effects over time. These findings support the potential predictive role of mindfulness and meditative practice in the reduction in stress levels in the short-term.

The practice of mindfulness is one of the strategies used for emotional intelligence reinforcement. The effectiveness of these training programs in preventing stress is closely related to the capacity for self-compassion, which allows the person to better manage emotions such as fear, anger, sadness, or doubt [58]. Furthermore, in the current epidemiological situation, these techniques are very useful to reduce symptoms associated with COVID-19, such as post-traumatic stress disorders, anxiety, or depression [59,60,61].

Several studies have been conducted in order to assess the effectiveness of mindfulness and meditation programs for healthcare professionals and healthcare professionals in training. Most of them have generally reported an improvement in coping strategies, a greater control of emotions, and a significant reduction in stress levels when these interventions have been carried out [62,63,64,65,66]. In a meta-analysis of 38 randomized clinical trials, Spinelli et al. quantified the effectiveness of mindfulness-based interventions on distress, well-being, physical health, and performance in qualified and trainee healthcare professionals [67]. The results of this review highlighted that the mindfulness training program had a small to moderate significant effect on stress at post-intervention (Hedge’s g = 0.52; 95% confidence interval (CI) 0.35 to 0.69) and follow-up time-points (Hedge’s g = 0.34; 95% CI 0.11 to 0.57). Fendel et al. performed a systematic review of clinical trials, whose objective was to evaluate the effectiveness of mindfulness-based intervention on stress levels among physicians [68]. They concluded that these interventions were associated with a significant medium reduction in stress in the between-group analysis of the randomized clinical trials (4 comparisons: standardized medium differences (SMD) = −0.55; 95% CI −0.95 to −0.14); *p* < 0.01; I^2^ = 24%) and a significant small reduction in stress in the pre–post analysis of all included studies (17 comparisons: SMD = −0.41; 95% CI −0.61 to −0.20); *p* < 0.001; I^2^ = 69%). However, in the systematic review carried out by Lomas et al., whose objective was to understand the value of interventions based on mindfulness and meditation in health professionals, several studies demonstrated no significant changes or worsening of the stress levels [69]. In this study, an improvement in PSQ total score, as well as PSQ-Tension, PSQ-Social, PSQ-Everyfun and PSQ-Satisfaction subscale scores was observed in the participants who received the standard 8-week MBRS training program. However, no changes were obtained in the PSQ-overload and PSQ-fear subscales scores, which indicates that these aspects are more resistant to change through interventions of this type.

The benefits of standard 8-week MBRS and self-compassion training programs on healthcare professionals have been widely demonstrated in the majority of the developed research to date [67]. In a randomized controlled clinical trial, Aranda et al. analyzed the effectiveness of this type of intervention to reduce stress levels and burnout in Primary Care professionals. Despite the limited response to the program, they suggest promoting mindfulness and self-compassion activities in the healthcare environment [41]. The effectiveness of training programs implemented for shorter periods of time, such as 3, 4, or 5 weeks, has been analyzed by different authors [70,71,72,73,74,75,76]. All of them concluded that these abbreviated interventions significantly reduced stress levels. In a systematic review by Gilmartín, all analyzed studies offered various types of brief mindfulness-based interventions and modalities to nurses/nursing students or physicians/medical students/resident intern specialists in hospital settings [77]. The effect of this type of program was associated with a significant improvement in provider well-being, especially in their stress levels. However, not enough studies have been carried out to provide solid evidence and compare the effectiveness of the abbreviated and standard MBRS training programs in healthcare professionals. As shown in the introduction, the scientific evidence comparing traditional and abbreviated mindfulness programs is scarce, but shows equivalent results between both treatments [26,49]. However, in this study, the data suggest that there was only a trend of improved perceived stress for EG1 after intervention, in contrast with the evidence of Zakiei et al. [78], who showed that improvements are observable within the first 3–4 weeks of any kind of treatment, and further improvements could also be expected till the end of an 8-week lasting intervention. In this case, only the standard MBRS training program had manage to improve the stress levels, but without the maintenance of its effects over time. The lower permeability of tutors and resident intern specialists to this type of intervention may be a possible explanation for the results obtained.

In this type of training program, the effects achieved in the short term are as important as their maintenance over time. Significant reductions in stress levels were maintained ranging from 2 months [71] to 1-year follow-up [79]. In a pilot study by Fortney et al., the maintenance of significant improvements in stress was demonstrated after a 9-month follow-up (change, −4.29; 95% CI −6.91 to −1.67; *p* = 0.002) [70]. After a 5-week mindfulness training program, Arneli et al., observed the maintained improvements in stress levels within EG from the end of the intervention to the 13-week follow-up (change, −6.14; CI 95% −7.88 to −4.44; *p* = 0.001) [69]. However, in this study, the effect of the standard MBRS training program on the stress levels in the post-test evaluation were not maintained over time. Furthermore, the mindfulness-related activity of the EGs participants after the interventions is unknown, so this is a heuristic hypothesis that should be tested in future research. In view of this result, a sustained practice of mindfulness over time may be necessary to achieve and maintain its effect on stress. In this sense, Fuertes et al. observed better maintenance of the effect of an 8-week training program on the stress level of participants who meditated regularly, with benefits persisting two years after completion [80].

This is one of the few studies published to date comparing the effects of a standard 8-week MBSR and MSC training program with an abbreviated 4-week one on stress levels in a group of tutors and resident intern specialists in Spain. However, these findings should be considered within the context of the study’s strengths and limitations. Among its main strengths were the use of validated instruments for the Spanish population which guarantees their validity and reduces the probability of information biases, the longitudinal methodology which allows determining causal relationships between the study variables, and the baseline stress levels were equivalent so all participants started from the same situation, as well as the evaluation of effect over time. On the other hand, the results obtained in the study may have been influenced by its own limitations, thus reducing its representativeness. Although each TU was considered as a different and independent cluster, randomly assigned to the CG or EGs in order to minimize the risk of contamination, statistically significant differences were observed between the three groups in age, type of professional, and time working in the Spanish National Health System, so this might be a cause of different interactions of professionals with the MBSR program, although no studies have been found in this regard in the literature consulted. As a consequence of the COVID-19 outbreak, the final sample size was lower than initially calculated, which may have influenced the results obtained. The baseline characteristics of participants who dropped out of the study were similar to those who completed it, so systematic selection bias is unlikely. Work obligations, family emergencies, shift changes, and illnesses were the reasons given for dropping out the study. Furthermore, an intention-to-treat analysis was performed in order to avoid this type of bias. The over representation of women, physicians, and Primary Care workers in the sample of the Spanish tutors and resident intern specialists in Family and Community Medicine or Nursing reduce the generalizing of study results. No theoretical–practical session of mindfulness or meditation was provided to the CG participants, and it was not possible to guarantee that they remained inactive during the fieldwork period, which could minimize the differences in the expected results when comparing this group with EGs. In the same way, the improvements obtained with the mindfulness and self-compassion treatment could be due to other variables, such as the interaction with other people during the sessions, since the passive CG lacks the opportunity for social interaction [78]. In addition, the practice of mindfulness or meditation by the EGs participants was not monitored after the post-test evaluation, so they could have practiced these or other techniques until the follow-up evaluation.

## 5. Conclusions

In order to provide quality healthcare, it is necessary to reduce the level of perceived stress faced by tutors and resident intern specialists in Family and Community Medicine and Nursing. Compared with an abbreviated program and no intervention., a standard 8-week MBSR training program produced significant improvements in PSQ total score as well as PSQ-Tension, PSQ-Social, PSQ-Everyfun and PSQ-Satisfaction subscale scores aimed at Primary Care professionals. However, the treatment effect was not maintained over time, and the 4-week version was not associated with significant changes in these variables. It is necessary to expand the exhaustive investigation of abbreviated programs that improve the psychological discomfort of these professionals, and analyze their cost-effectiveness so that the SNS can include these programs in its policies, with a guarantee of adherence and long-term profitability.

## Figures and Tables

**Table 1:** Baseline characteristics of participants.

Variable	Total *n* = 112	CG *n* = 51	EG1 *n* = 24	EG2 *n* = 37	*p*-Value	η^2^
Age (years): mean ± SD	41.61 ± 12.61	40.34 ± 13.22	47.66 ± 13.67	35.73 ± 12.04	<0.001	0.109 *
Sex: *n* (%)						
Male	26 (23.21)	11 (21.57)	6 (25.00)	9 (24.32)	0.978	0.016 **
Female	86 (76.79)	40 (78.43)	18 (75.00)	28 (75.68)		
Occupation: *n* (%)						
Physician	95 (84.82)	41 (80.39)	20 (83.33)	34 (91.89)	0.165	0.146 **
Nurse	17 (15.18)	10 (19.61)	4 (16.67)	3 (8.11)		
Professional type: *n* (%)						
Tutor	50 (44.64)	24 (47.06)	15 (62.50)	11 (29.73)	<0.001	0.317 **
Resident	62 (55.36)	27 (52.94)	9 (37.50)	26 (70.27)		
Workplace: *n* (%)						
Health Center	95 (84.82)	40 (78.43)	22 (91.67)	33 (89.19)	0.217	0.135 **
Hospital	17 (15.18)	11 (21.57)	2 (8.33)	4 (10.81)		
Work experience (years): mean ± SD	12.88 ± 13.15	13.13 ± 12.95	19.49 ± 13.91	8.91 ± 11.06	<0.001	0.117 *

Abbreviations: CG: Control Group; EG1: Experimental Group, 4 weeks; EG2; Experimental Group, 8 weeks; SD: standard deviation. * η^2^; ** Contingency Coefficient.

**Table 2:** Inter-group comparison of PSQ at different evaluation points, using one-way ANOVA.

Evaluation	Outcome	CG		EG1		EG2		F	*p*-Value	η^2^
		Mean	DS	Mean	DS	Mean	DS			
Pretest	PSQ Tension–Instability–Fatigue	**23.825**	**4.316**	22.846	4.749	23.159	4.674	0.636	0.531	0.008
	PSQ Social Acceptance of Conflicts	13.778	3.250	13.897	3.705	13.937	3.136	0.038	0.963	0.000
	PSQ Energy and Fun	13.746	2.609	13.231	2.146	13.191	2.429	0.959	0.385	0.012
	PSQ-Overload	10.032	1.565	10.513	1.315	10.159	1.677	1.178	0.311	0.014
	PSQ Self-Fulfillment Satisfaction	6.809	1.378	6.744	1.332	6.460	1.280	1.184	0.309	0.014
	PSQ Fear and Anxiety	3.984	1.277	4.051	1.169	4.349	1.138	1.596	0.206	0.019
	PSQ Total	69.250	10.910	68.430	12.180	68.740	11.650	0.100	0.905	0.001
Post-test	PSQ Tension–Instability–Fatigue	23.750 *	5.501	21.357	6.696	20.195 *	5.016	4.972	0.008	0.075
	PSQ Social Acceptance of Conflicts	14.536 *	4.191	13.250	4.766	12.146 *	3.403	4.086	0.019	0.063
	PSQ Energy and Fun	12.911 *	3.354	11.536	3.328	10.854 *	3.062	4.989	0.008	0.076
	PSQ-Overload	10.732	2.416	11.393	2.439	10.171	2.397	2.142	0.122	0.034
	PSQ Self-Fulfillment Satisfaction	6.607 *	1.775	5.893	1.873	5.122 *	1.327	9.440	<0.001	0.134
	PSQ Fear and Anxiety	3.929	1.373	4.143	1.484	3.830	1.181	0.463	0.631	0.008
	PSQ Total	69.660 *	15.230	65.210	17.590	59.900 *	12.810	4.970	0.008	0.075
Follow-up	PSQ Tension–Instability–Fatigue	24.039	6.591	21.375	6.889	21.324	5.318	2.568	0.081	0.045
	PSQ Social Acceptance of Conflicts	14.745	4.462	13.833	5.346	12.540	3.114	2.837	0.063	0.049
	PSQ Energy and Fun	12.529	3.596	11.750	3.542	11.216	2.678	1.733	0.182	0.031
	PSQ-Overload	11.059	2.525	10.833	2.914	10.459	2.479	0.572	0.566	0.010
	PSQ Self-Fulfillment Satisfaction	6.686 *	2.131	6.417	2.104	5.513 *	1.539	4.006	0.021	0.068
	PSQ Fear and Anxiety	4.000	1.456	4.208	1.532	3.784	1.228	0.685	0.506	0.012
	PSQ Total	70.390	16.950	66.040	19.570	62.450	13.200	2.531	0.084	0.044

* *p*-value < 0.05 in post-hoc analysis (Bonferroni test) between CG and EG2. Abbreviations: CG: Control Group; EG1: Experimental Group, 4 weeks; EG2: Experimental Group, 8 weeks; SD: Standard Deviation; η^2^: Squared Eta Coefficient; PSQ: Perceived Stress Questionnaire.

**Table 3:** Intra-group comparison of PSQ at the same evaluation point, using ANOVA for repeated measures.

Group	Outcome	Pretest		Post-Test		Follow-Up		MS	F	*p*-Value	η^2^
		Mean	DS	Mean	DS	Mean	DS				
CG	PSQ Tension–Instability–Fatigue	24.273	3.660	23.485	5.723	23.818	6.217	5.162	0.283	0.755	0.009
	PSQ Social Acceptance of Conflicts	14.000	3.419	14.758	4.451	14.364	4.227	4.737	0.748	0.477	0.023
	PSQ Energy and Fun	14.091 *	2.542	13.181 *	3.548	12.576	3.410	19.192	4.780	0.012	0.130
	PSQ-Overload	10.212	1.453	10.879	2.434	10.939	2.573	8.727	3.662	0.065	0.103
	PSQ Self-Fulfillment Satisfaction	6.818	1.530	6.697	1.845	6.727	2.212	0.131	0.069	0.934	0.002
	PSQ Fear and Anxiety	3.879	1.317	3.939	1.345	4.000	1.346	0.121	0.116	0.891	0.004
	PSQ Total	69.250	10.910	69.660	15.230	70.390	16.950	2.303	0.022	0.978	0.001
EG1	PSQ Tension–Instability–Fatigue	22.118	4.386	21.588	6.423	20.941	6.590	5.902	0.369	0.694	0.023
	PSQ Social Acceptance of Conflicts	13.353	3.622	13.353	4.015	14.118	4.885	3.314	0.518	0.601	0.031
	PSQ Energy and Fun	12.823 *	1.846	11.235 *	3.093	11.353	3.517	13.314	3.758	0.034	0.190
	PSQ-Overload	10.647	1.169	11.294	2.312	10.706	2.995	2.176	0.839	0.441	0.050
	PSQ Self-Fulfillment Satisfaction	6.588	1.004	5.765	1.562	6.412	2.293	3.196	2.498	0.098	0.135
	PSQ Fear and Anxiety	3.941	1.088	4.000	1.275	4.294	1.359	0.608	0.967	0.391	0.057
	PSQ Total	68.430	12.180	65.210	17.590	66.040	19.570	13.549	0.156	0.857	0.010
EG2	PSQ Tension–Instability–Fatigue	23.167 *	4.146	20.500 *	5.626	21.083	4.880	47.167	3.505	0.038	0.132
	PSQ Social Acceptance of Conflicts	13.917	3.035	12.583	3.106	12.667	3.185	13.389	2.134	0.130	0.085
	PSQ Energy and Fun	13.042 *	2.349	10.833 *	3.046	11.333	2.408	32.181	9.471	<0.001	0.292
	PSQ-Overload	10.542	1.587	10.625	2.651	10.292	2.216	0.722	0.206	0.815	0.009
	PSQ Self-Fulfillment Satisfaction	6.542 ^$^	1.503	5.250	1.189	5.458 ^$^	1.587	11.542	11.479	<0.001	0.333
	PSQ Fear and Anxiety	4.292 *	1.083	3.958 *	1.122	3.667	1.341	2.347	3.242	0.048	0.124
	PSQ Total	68.740 *	11.650	59.900 *	12.810	62.450	13.200	393.431	4.587	0.015	0.166

* *p*-value < 0.05 in post-hoc analysis (Bonferroni test) between pretest and post-test. ^$^ *p*-value < 0.05 in post-hoc analysis (Bonferroni test) between pretest and follow-up. Abbreviations. SD: Standard deviation; MS: Mean Square; η^2^: Squared Eta Coefficient; CG: Control Group; EG1: Experimental Group, 4 weeks; EG2: Experimental Group, 8 weeks; PSQ: Perceived Stress Questionnaire.

**Table 4:** Comparison between groups in post-test and follow-up scores, controlling pretest scores, using ANCOVA.

Evaluation	Outcome	Source	Type III Sum of Square	df	MS	F	*p*-Value	η^2^
Post-test	PSQ Tension–Instability–Fatigue	Pretest PSQ Tension–Instability–Fatigue	536.007	1	536.007	20.144	<0.001	0.181
		CG/EG1/EG2	172.882	2	86.441	3.249	0.043	0.067
		Error	2421.378	91	26.609			
	PSQ Social Acceptance of Conflicts	Pretest PSQ Social Acceptance of Conflicts	352.796	1	352.796	30.146	<0.001	0.249
		CG/EG1/EG2	114.431-	2	57.216	4.889	0.010	0.097
		Error	1064.951	91	11.703			
	PSQ Energy and Fun	Pretest PSQ Energy and Fun	251.198	1	251.198	34.118	<0.001	0.273
		CG/EG1/EG2	52.589	2	26.294	3.571	0.032	0.073
		Error	669.991	91	7.363			
	PSQ Overload	Pretest PSQ Overload	92.181	1	92.181	19.326	<0.001	0.175
		CG/EG1/EG2	17.248	2	8.624	1.808	0.170	0.038
		Error	434.053	91	4.770			
	PSQ Self-Fulfillment Satisfaction	Pretest PSQ Self-Fulfillment Satisfaction	41.923	1	41.923	21.435	<0.001	0.191
		CG/EG1/EG2	35.168	2	17.584	8.991	<0.001	0.165
		Error	177.977	91	1.956			
	PSQ Fear and Anxiety	Pretest PSQ Fear and Anxiety	40.719	1	40.719	34.263	<0.001	0.274
		CG/EG1/EG2	1.870	2	0.935	0.787	0.458	0.017
		Error	108.146	91	1.188			
	PSQ Total	Pretest PSQ Total	4255.714	1	4255.714	26.222	<0.001	0.224
		CG/EG1/EG2	1676.611	2	838.305	5.165	0.008	0.102
		Error	14,768.788	91	162.294			
Follow-up	PSQ Tension– Instability-Fatigue	Pretest PSQ Tension–Instability–Fatigue	207.608	1	207.608	6.241	0.015	0.073
		CG/EG1/EG2	77.888	2	38.944	1.171	0.315	0.029
		Error	2628.146	79	33.268			
	PSQ Social Acceptance of Conflicts	Pretest PSQ Social Acceptance of Conflicts	279.122	1	279.122	20.781	<0.001	0.208
		CG/EG1/EG2	43.832	2	21.916	1.632	0.202	0.040
		Error	1061.119	79	13.432			
	PSQ Energy and Fun	Pretest PSQ Energy and Fun	200.447	1	200.447	23.134	0.001	0.227
		CG/EG1/EG2	2.880	2	1.440	0.166	0.847	0.004
		Error	684.514	79	8.665			
	PSQ Overload	Pretest PSQ Overload	48.077	1	48.077	8.358	0.005	0.096
		CG/EG1/EG2	12.184	2	6.092	1.059	0.352	0.026
		Error	454.429	79	5.752			
	PSQ Self-Fulfillment Satisfaction	Pretest PSQ Self-Fulfillment Satisfaction	63.076	1	63.076	19.201	<0.001	0.196
		CG/EG1/EG2	13.817	2	6.908	2.103	0.129	0.051
		Error	259.523	79	3.285			
	PSQ Fear and Anxiety	Pretest PSQ Fear and Anxiety	26.231	1	26.231	17.133	<0.001	0.178
		CG/EG1/EG2	6.806	2	3.403	2.223	0.115	0.053
		Error	120.954	79	1.531			
	PSQ Total	Pretest PSQ Total	2647.821	1	2647.821	12.098	0.001	0.133
		CG/EG1/EG2	621.887	2	310.943	1.421	0.248	0.035
		Error	17,290.561	79	218.868			

Abbreviations. df: Degrees of Freedom; MS: Mean Square; η^2^: squared eta coefficient; CG: Control Group; EG1: Experimental Group, 4 weeks; EG2: Experimental Group, 8 weeks; PSQ: Perceived Stress Questionnaire.
